# Visceral Leishmaniasis in Traveler to Guyana Caused by *Leishmania siamensis*, London, UK

**DOI:** 10.3201/eid2408.172147

**Published:** 2018-08

**Authors:** Jérôme Depaquit, Matthieu L. Kaltenbach, Frédérick Gay

**Affiliations:** Reims Champagne-Ardenne University, Rheims, France (J. Depaquit, M.L. Kaltenbach);; Maison Blanche Hospital, Reims (J. Depaquit);; Pitié-Salpêtrière Hospital, Paris, France (F. Gay);; Sorbonne University, Paris (F. Gay)

**Keywords:** visceral leishmaniasis, Leishmania siamensis, L. martiniquensis, parasites, traveler, Guyana, London, UK

**To the Editor:** In a case report of visceral leishmaniasis in a traveler returning from Guyana, Polley et al. identified *Leishmania siamensis* as the causative agent (*1*). However, we believe that the parasite responsible for this infection has been misidentified. Classification of parasites formerly identified as *L. siamensis* has recently been revisited (*2*) after description of a new species (*L. martiniquensis*) from the West Indies (*3*). All previously described *L. siamensis* strains, except 1, are now reported as *L. martiniquensis*. Their rDNA internal transcribed spacer 1 sequences are still deposited in GenBank under the name *L*. *siamensis*. The exception, reported from Thailand (GenBank accession no. JX195640), is the only known *L. siamensis* sample to date.

New analysis of *Leishmania* (*Mundinia*) sequences available in GenBank and of *L. infantum* showed no variability in *L. martiniquensis*, including the sequence (GenBank accession no. LT577674) reported by Polley et al. (*1*), and sequence divergence when compared with *L. siamensis* (32.4%), a *Leishmania* sp. from Ghana (32.3%) (*4*), *L. enrietti* (30.6%), and *L. infantum* (43.6%). *L. martiniquensis* has been reported worldwide (Florida, West Indies, central Europe, and Southeast Asia). However, *L. siamensis* has been reported only once (in Thailand).

If one considers possible quiescence of the parasite, and that the patient was from Guyana, migrated to the United Kingdom in 1967, and had a relevant travel history, including visits to France (2003), Ghana (2005), Caribbean Grenada (2012), and Guyana (2012 and 2013), the geographic origin of this infection is unknown. Moreover, the mode of transmission of *L. martiniquensis* is not yet clearly defined. In contrast to the report of Polley et al. (*1*), although the genus *Sergentomyia* could play a role in some foci of leishmaniasis, it has never been recorded in the Americas (*5*).
